# The Spread of Ideas in a Network—The Garbage-Can Model

**DOI:** 10.3390/e23101345

**Published:** 2021-10-14

**Authors:** Dorota Żuchowska-Skiba, Maria Stojkow, Malgorzata J. Krawczyk, Krzysztof Kułakowski

**Affiliations:** 1 Department of Humanities, AGH University Science and Technology, Aleja Adama Mickiewicza 30, 30-059 Kraków, Poland; zuchowska@agh.edu.pl (D.Ż.-S.); stojkow@agh.edu.pl (M.S.); 2 Department of Physics and Computer Science, AGH University Science and Technology, Aleja Adama Mickiewicza 30, 30-059 Kraków, Poland; malgorzata.krawczyk@agh.edu.pl

**Keywords:** garbage can model, Axelrod model, evolving ideas

## Abstract

The main goal of our work is to show how ideas change in social networks. Our analysis is based on three concepts: (i) temporal networks, (ii) the Axelrod model of culture dissemination, (iii) the garbage can model of organizational choice. The use of the concept of temporal networks allows us to show the dynamics of ideas spreading processes in networks, thanks to the analysis of contacts between agents in networks. The Axelrod culture dissemination model allows us to use the importance of cooperative behavior for the dynamics of ideas disseminated in networks. In the third model decisions on solutions of problems are made as an outcome of sequences of pseudorandom numbers. The origin of this model is the Herbert Simon’s view on bounded rationality. In the Axelrod model, ideas are conveyed by strings of symbols. The outcome of the model should be the diversity of evolving ideas as dependent on the chain length, on the number of possible values of symbols and on the threshold value of Hamming distance which enables the combination.

## 1. Introduction

Nowadays, the concept of ideas often appears in social sciences in the context of innovation [1,2], with a strong emphasis on their objective character. In such approaches, the relationship between the idea and the objects or solutions to which it applies clearly takes a center stage. However, this point of view ignores the issue of innovation and the complex processes involved in the formation of ideas. Rico Sneller [3] suggests redefining ideas in a way that does away with the traditional division into subject and object, takes into account the existence of synchronicity underlying the birth of certain ideas in different environments and recognizes a certain degree of opacity. From this perspective, ideas do not merely serve as practical solutions or useful tools, but rather provide us with a means of overcoming human problems, whether it be in the fields of science, philosophy, religion or society. This approach allows for the immediate implementation of plans connected with e.g., the development and improvement of a weapon, maximizing a company’s profit, increasing scientific research and results, etc. In other words, ideas convey a disposition to break through, whether in terms of thinking or acting, an impasse [3]. This can take the form of an invention, an algorithm, a procedure, a routine or even a story. Formally, this process involves selecting a certain subset of elements out of a larger set of solutions.

This approach, by no means complete, constitutes a mathematical model. When adopting this path, we make inferences on certain phenomena and processes in social life from their mathematical representations [4]. We underline the fact that a model contains no justification for this or any other representation; it is just assumed. Similarly, any interpretation of the model’s results cannot be assessed within the model itself. Both assumptions and interpretation are open ends of the path of reasoning.

In addition, assumed are the dynamics of the idea:

First—the process of integrating/creating a complementary idea in response to the act of searching. This mechanism is supposed to work, if the current distance between existing ideas is not too great. The distance is measured in the space of states; similar objects are closer. The space of states is either an axis, or a network. There is also another condition of integration: the communities in which particular ideas evolve should be in contact. In our modelling, this contact is externally switched on.

Second—the role of randomness (coincidences) in the formation of an idea—here we will examine the issue of randomness in selected examples illustrating the dynamics of an idea. At the beginning, we will focus on the phenomenon of synchronicity paired with ideas. This term was coined by Carl Gustav Jung [5] (p. 8) and is defined as the occurrence of two phenomena in parallel lines, such as an event or psychic fields, where the observer and the commonly accepted meaning are not in parallel [6] (pp. 59–62), [7].

Thirdly, to clarify the role of chance in decision making we will focus on the ‘garbage can’ model [8] of organizational choice. According to this model, all organizational decisions taken are the outcome of a random process, modeled as collisions between problems, answers and means. We intend to explore the role of randomness/coincidences in selected examples involving the dynamics of ideas.

Our goal is to describe the following effect. In two communities, initially isolated from one another, ideas evolve towards two distinct integrated forms. At some point in this process, contact between the communities is enabled. Two kinds of outcome are possible. If in the integrated version the two communities are not too distant at the moment of contact, the process of integration continues towards one final version of the idea. Otherwise, the process continues as two separate phenomena in the two communities in isolation; the contact was established at too late a date. This is our sequence of cause and effect. To identify examples of this sequence in observed phenomena, there should be an opportunity to repeat the process by manipulating the time when contact is enabled. However, such experiments with ideas are not repeatable; for an account of cause-effect relations we refer to [9]. Examples can only be gathered when integration between ideas proceeds in two communities in mutual contact, or when the two ideas remain isolated from one another.

Such examples will be presented in the next part of the article. In Section 3, two formulations of the model are given: one analytical and one computational. The first one is deterministic and offers closed mathematical expressions, but is greatly simplified. The second contains some degree of randomness, as it relies on Monte Carlo simulations. It is less transparent, but more directly related to the picture of model dynamics outlined above. Section 4 is devoted to discussion. There, the examples provided in Section 2 are scrutinized again, with the comments on the evolution, fusion or split of the related ideas. The final section is devoted to conclusions.

## 2. Materials and Methods

Ideas can be found either apart from or complementing each other, or as compilation of manufactured ideas and new original solutions. Agriculture was invented in different places at around the same time. A similar pattern can be observed in the case of new sciences. For example, the science that came to be known as “biology” was pioneered simultaneously around 1800 by Treviranus, Beddoes, Lamarck and Burdach [3,10]. Below are some examples that illustrate the dynamics of ideas:

[A] The concept of self-organized criticality was formulated in 1987 by Per Bak and his co-authors [11] on the basis of a study of avalanches on a sandpile. A meeting between Bak and Kim Sneppen, described in [12], resulted in an enriched model based on the idea of modifying the weakest element in the system. The combined version is known as the Bak-Sneppen model [13], which became a key approach in self-organized criticality [14]. From our point of view, the case is an example of a successful fusion of two complementary ideas.

[B] During the so-called Leipzig Debate in 1519, a final and irrevocable schism occurred between the Catholic and Protestant churches [15]. This outcome was a consequence of different positions taken on a fundamental issue: the legitimacy of papal authority. Luther’s doctrine of Sola scriptura could not be reconciled with Pope Pius II’s decree of 1460 declaring that ‘even an appeal to a church council was heretical’ [16].

[C] In 1971, the literary critic Tzvetan Todorov analyzed the text of *Les liaisons dangereuses* by Pierre Choderlos de Laclos and published a kind of grammar of the rules governing the plot [17]. Thirty years later the psychologist Colin Martindale discovered [18] that these rules can be derived from Heider’s theory of structural balance in interpersonal relations [19]. Martindale [18] (p. 400) concluded: ‘Had Todorov been aware of Heider’s model, he would have seen this immediately’. Models of Heider balance can be further interpreted in detail within the Axelrod model, as shown in Section 4.

[D] The classical boundary between images of particles and waves had to be abandoned in the first half of the twentieth century, giving way to what is known as wave-particle duality [20]. Prominent physicists at the time approached the issue as follows: “Is light a wave or a shower of photons? Is a beam of electrons a shower of elementary particles or a wave? These fundamental questions are forced upon physics by experiment” [21] (p. 334). This example adds to the list of examples where ideas were successfully merged, despite having been separated for a long period of time.

[E] Belief in the superiority of the spiritual over the material world, the concept of God as the first cause and purpose of the universe, the idea that the material world is saturated with spirit, an indifference to worldly things, and the exclusion of the values of sensual and rational cognition—today these elements are associated with early Christianity. In actual fact, what is today treated as a consistent doctrine was a blend of ideas adopted from earlier philosophical movements: Platonism, as borrowed by Augustine, Aristotelianism, as adapted by Aquinas, stoicism, cynicism and skepticism, respectively [22] (p. 127). A glance at ancient mythologies reveals an even richer repository of ideas [23,24].

[F] In the first half of the twentieth century, two variants of quantum mechanics were developed—Werner Heisenberg’s matrices [25] and the differential equations of Erwin Schrödinger [26]. The aim of both approaches was to describe the same class of experiments. However, their equivalence remained an open issue until the publication of John von Neumann [27]. As was concluded in [28], it is now clear that both formalisms “must always yield the same empirical predictions”; in this sense they, too, constitute a unified concept.

[G] As all of us know, the British and American languages have the same root; yet they differ [29,30]. Whether they are going to be identical, is an open issue [31]. A further partition of English into local mixtures [32] seems more probable. These splits are expected to be correlated with related splits of identity [33].

## 3. Results

### 3.1. The Deterministic Picture

The first, analytical formulation draws from the Hegselmann–Krause (HK) model of opinion dynamics [34]. When adopted for our purposes, it can be described as follows: ideas are represented by points on an axis. The integration process is executed through the mutual attraction of interacting ideas. Provided that these ideas are distributed with initial density *ρ*(*y*) over a range between *a* and *b* on the axis, the dynamics of an idea at point *x* is given by the following equation:

(1) $$\frac{dx}{dt}=\int_{a}^{b}dy(y-x)\rho (y)$$

where *a* ≤ *x* ≤ *b*. This means that an idea at point *x* is attracted by an idea *y* with intensity *y* − *x*.

The rationale of this setting is that opinions that are more remote from ours, if accepted, force us to modify our own opinions to a greater extent. We note that according to Equation (1) if *y* > *x*, *x* is pushed upwards, and if *y* < *x*, *x* is reduced.

In the case of homogeneous initial distribution *ρ*(*y*) = 1/(*b* − *a*), this equation can be easily solved. It appears that distribution *ρ* remains homogeneous, its center of mass, initially equal to (*a + b*)/2, remains constant in time, and its width, initially equal (*b − a*), shrinks in time as (*b* − *a*) exp(−*t*).

**Figure 1:**
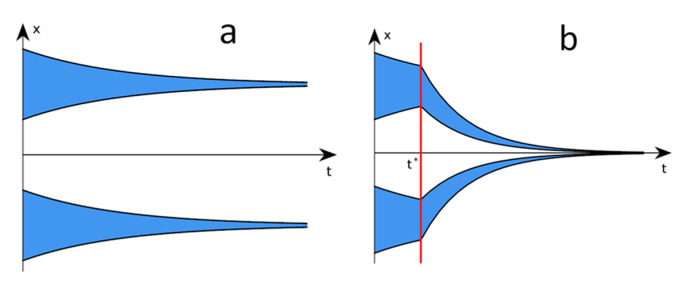
(**a**) Two bands evolve without contact. These bands remain concentrated around different initial values. Their widths decrease as exp(−*t*)/2. (**b**) At time t*, mutual contact between the communities is enabled if their distance is smaller than *H*. For a higher value of *H*, the entire range of opinions varies in width as exp[−2(*t* − t*)].

In Figure 1a we present the time evolution of two such bands 1 and 2, with exemplary values *a*(1) = −3/4, *b*(1) = −1/4, *a*(2) = 1/4 and *b*(2) = 3/4. Two bands evolve without mutual contact; they remain concentrated around values ±1/2, and their widths decrease as exp(−*t*)/2. The distance between their boundaries increases as *D*(*t*) = 1 − exp(−*t*)/2. Now, suppose that at time t* a contact becomes possible between ideas which differ by no more than *H*. Such a distance dependent interaction is a key element of the Hegselmann–Krause model. It is clear that if *D*(t*) > *H*, then contact is not effective and the ideas from different bands remain independent.

In a case where *H* is only slightly greater than *D*(t*), density *ρ* is no longer homogeneous and the related equations are more complex. For the purposes of completeness, we solve the case when *H* > 1 + exp(−t*)/2. Then, all ideas interact and the result is simple again. It appears that for the initial positions of the bands as given above, all the trajectories tend to zero. Namely, for the parameters as in Figure 1 the entire range of ideas varies in width as:

(2) W(t)=[1+exp(−t*)/2]exp[−2(t−t*)]

and tends to zero for a long period of time *t*. This means that the unification is complete. The related plots are shown in Figure 1b.

### 3.2. The Stochastic Picture

The second, computational formulation is based on three concepts: (i) temporal networks [35], (ii) the Axelrod model of culture dissemination [36], and (iii) the garbage can model of organizational choice [8]. The use of the concept of temporal networks makes it possible to show the dynamics of idea-spreading processes in networks, thanks to an analysis of contacts between agents in networks. The Axelrod culture dissemination model takes into account the importance of cooperative behavior in the dynamics of ideas disseminated in networks. In the garbage can model, solutions to problems are reached as an outcome of sequences of pseudorandom numbers. The origin of this model is Herbert Simon’s [37] theory of bounded rationality. According to Simon, human decisions do not maximize utility; rather, they are narrowed by the limitations of time and knowledge of decision-makers [37].

The representation of ideas is taken from the Axelrod model [36]. In a set of *N* ideas, each idea is represented by a string of cells, with each cell containing a symbol. The string length is denoted as *F*, and each symbol can take one of *q* values. The space between states is then *F*-dimensional. The distance between strings is defined as the Hamming distance, i.e., the number of cells where the symbols are different.

**Figure 2:**
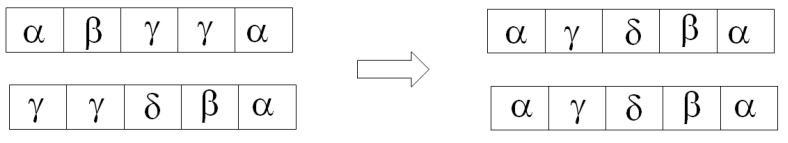
In cases where all the symbols in both strings are different, they remain unchanged. However, for *dc* = *F* (here *F* = 5) and if in both strings the symbols are the same in at least one cell, the strings unify, i.e., all the symbols in the corresponding cells are set equally. Such a case is presented here, as the symbols in the last cells of both strings on the left are α. On the right the strings are shown after their unification.

The dynamics of the model is as follows. In each time step, a pair of strings {*j*,*k*} are selected randomly, and the distance *d*(*j*,*k*) between them is calculated. Once this distance is smaller than prescribed value *dc*, both strings unify: the symbols are set at equal in all their cells, with values adopted from *j* or *k* with the same probabilities. A flowchart for a one-time step is shown in Figure 2. The randomness of the pair selection is related to the garbage can model.

We note that the process is equivalent to an aggregation of strings distributed in a discrete space of states. Then, the condition of merging of two strings is the same: the inter-string distance in this space is smaller than *dc*. It should be clear, however, that also in this representation the mutual contact between groups is disabled or enabled from outside. The latter change reflects an exchange of information, which does or does not occur.

Here once more we will endeavor to demonstrate that if ideas evolve in separated communities for a sufficiently long period of time, they can remain different from one another even when contact is enabled. This task is completed as follows. We simulate the dynamics in parallel in *K* different groups, selecting both strings of each pair exclusively from the same group. After t* time steps, the strings of a pair can also be taken from different groups. Suppose that in the first stage (t* time steps) the strings are unified within each group. The question is: do they unify in the second stage?

In a simple case, the question can be dealt with by means of an inspection. Suppose that *F* = *dc*. Then, the strings unify even if they have all but one cell occupied by different values of symbols. In other words, the only situation when they do not unify is if all values of symbols in the corresponding pairs of cells are different. The probability that two strings do not unify is then *P* = (1 − 1/*q*)*^F^*. Consider the case *K* = 2, where all strings within each group are unified during the first t* time steps. The distance between the strings in different groups is equal to *F* with probability *P*, which decreases with *F* and increases with *q*.

**Figure 3:**
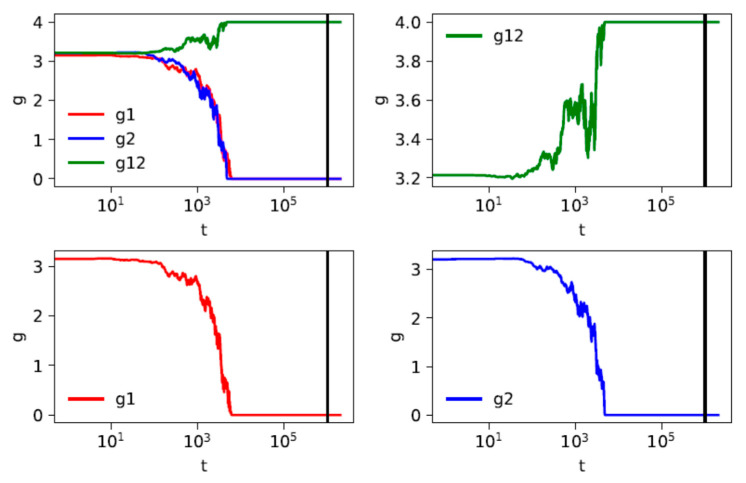
An example of a numerical solution obtained using the computational method for two groups, each of *N*/2 = 50 strings. The mean distance between strings within groups (*g*1 and *g*2, lower panels), between groups (*g*12, upper right panel) and taken as a whole (upper left panel) versus time. The parameters are as follows: *F* = 4, *q* = 5, *dc* = 4, t* = 10^6^; the latter parameter is marked as a vertical black line.

**Figure 4:**
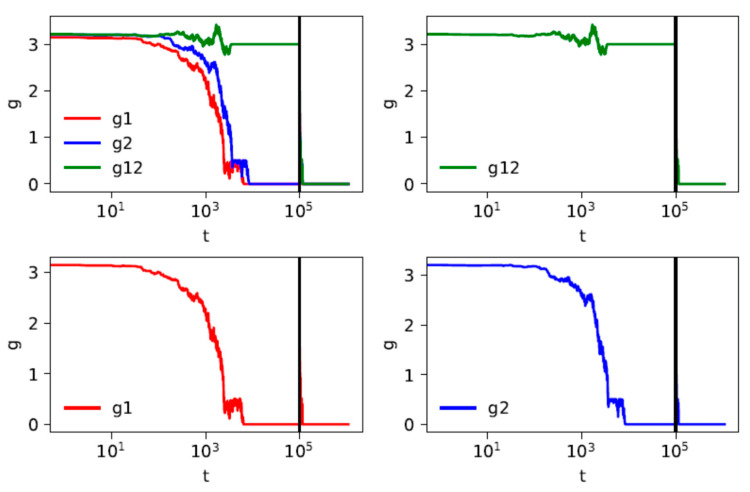
An example of a numerical solution obtained using the computational method for two groups, each of *N*/2 = 50 strings. The mean distance between strings within groups (*g*1 and *g*2, lower panels), between groups (*g*12, upper right panel) and as a whole (upper left panel) versus time. The parameters are: *F* = 4, *q* = 5, *dc* = 4, t* = 10^5^; the latter parameter is marked as a vertical black line.

In Figure 3 and Figure 4 present we two examples of the time dependence of mean distances within two groups and between the groups. In each case the individual trajectories are shown of a system of *K* = 2 groups, *N/K* = 50 strings in each group. The averaging is performed over the pairs of strings in one system, and not over a number of different systems; hence, visible fluctuations. The reason of such presentation is that the aim is to demonstrate two different scenarios:

For selected values of parameters *F* = 4, the maximal distance is equal to 4. If the mean distance *g*12 between groups is equal to 4, as shown in Figure 3, then all distances between the groups are equal to 4. Furthermore, as *dc* = 4, the distances between the strings in different groups cannot be reduced by the time evolution. In the case shown in Figure 3, strings are fully unified within each group, but the distances between the strings in different groups are maximal. Therefore, the groups will not be unified, and *g*12 remains equal to 4 even for *t* > t*.

In the case shown in Figure 4 the distances between elements of different groups at t* are less than 4, which means that they can be reduced once the contact between the groups has been enabled. Indeed, these distances are reduced to zero soon after t*. This means that all the strings are fully unified in one state within the system as a whole, and for *t* > t* the intergroup distance *g*12 is reduced to zero. The situations presented in Figure 3 and Figure 4 correspond to those shown in Figure 1a,b in the analytical approach.

To gain more insight into these results, we have evaluated the final diversity of the node states, taking into acount the model parameters. A conventional measure of diversity is the Shannon entropy [38,39].

(3) $$S=-\sum_{i}^{}p(i)\ln p(i)$$

where *p*(*i*) is the probability of *i*-th state of the string. As is already known, *S* acquires its maximal value when all states are equally probable; then *Smax* = ln(*Ω*), where *Ω* is the number of all states. Here *Ω* = *q^F^*, then *Smax* = *F* ln(*q*). For the results shown below in Table 1 we assigned an average value of *s* = *S/Smax* instead of *S*, to extract the dependence on *F* and *q* which comes from different values of *Ω*. This average is calculated over 50 single trajectories of the system. In particular, for a state where all strings happen to be identical in both groups, its contribution to the average entropy is zero, despite the fact that such a state can be different for different simulations.

The results shown in Table 1 together with those for t* = 10^3^ (not shown here) allow us to draw the following conclusions:

- A change in *q* mostly changes the normalization constant *Smax*; after excluding this dependence, <*s*> remains almost the same for different *q*. In particular, the <*s*> values in the first two rows in the table are similar to those in the next two rows. The same can be observed in the four last rows;
- On the contrary, the consequences of change in *F* cannot be reduced to a variation of the normalization constant. When *F* increases from 3 to 4, <*s*> is reduced more than twice;
- The results clearly depend on the threshold *dc*. For *dc* = *F*, entropy is about five times less than for *dc* = *F* − 1. More generally, with smaller *dc* the unification is more limited, and the diversity of the states is greater.

## 4. Discussion

The two models presented in Section 3 are designed in such a way so as to refer to the same phenomenon in which a collection of ideas is integrated, or—alternatively—remain separated. The concept (to avoid the term ‘idea’ at this point) common to both is the distance between ideas. We should underline the fact that this concept makes sense only within each example, and cannot be used to measure, say, the distance between *Sola scriptura* and a stream of photons. In addition, it could be tempting to treat the real number (in binary or decimal form) in the first model or a string of symbols in the second as a text describing an idea. However, such a conversion needs a specific language, where a negation is equivalent to a change in all the symbols in a sentence. This means, that the two models are not equivalent.

The models differ also in another regard. The first, analytical model, is purely deterministic: its outcome depends solely on the initial state and on time t*. The second is stochastic: its outcome depends on the pseudorandom numbers used in the simulation, which determines the order of selection of pairs of strings. Hence the numerical results should be averaged over a number of runs. In the first model, an idea is represented by a single real number, and in the second by *F* integers. We will endeavor to classify the examples provided in Section 2 and comment on the aspects described above.

[A] The Bak-Sneppen model can be viewed as a tangle of two algorithms, each with its own input and output. The first algorithm operates according to the idea of an avalanche, while the second is based on notion of the weakest element. These two are not alternatives to each other, but rather complementary. In addition, the Bak-Sneppen model is just one of a number of realizations of self-organized criticality (for a review, see [14]). As a consequence, we can treat this model as a string of symbols, each composed of two shorter strings. It is likely that this composition is not unique, hence the concept of diversity makes sense. In this case, the diversity increases, and the entropy is a sum of components, related to the shorter strings.

[B] The Leipzig debate was only one event in time sequence, with the famous declaration of the 95 Theses rightly considered to be the primary cause. In actual fact, the initial state in this sequence is Catholic doctrine itself. However, in this example the initial state is the potential split in the Church, with two possible outcomes: unity restored or an ultimate schism. A number of seemingly accidental factors emerged, such as Johann Eck’s strategy of including the legitimacy of papal authority in the debate. When the sequence is arranged in this way, there was no chance of a compromise. When modeled, the issue is solved by one cell in the string, no matter what the values of the symbols in the other cells are. Effectively, *F* = 1. The HK approach is more straightforward to use: we can represent numbers through their binary form, and then compare only first digits (first cells in both strings) to obtain the answer.

[C] What was stated by Todorov is just one item in a long list of ideas that have been invented more than once and whose authors were unaware of the existence of each other. Time t* is the moment when they are identified as equivalent. Since then, the integration was immediate. Actually, the concept of Heider balance has been used in many different models and contexts [40,41,42]. This is an indication that the discrete approach works better. A new aspect of the issue can be represented as growing value of the parameter *F*.

This case can be further explored to exemplify the role of model assumptions as the values of symbols in the related string. The Heider balance has been discussed in 2005 independently by Tibor Antal et al. [43] and by one of present authors, with coworkers [44]. In these works, three cells (*F* = 3) of the model formulation can be distinguished easily: (i) continuous (α) or discrete (β) character of interpersonal relations, (ii) local (α) or global (β) coupling between the variables, and (iii) synchronous (α) or asynchronous (β) dynamics. Within this scheme, the approach [43] can be encoded as [βαβ] and [βββ] (as there are two versions of the model in [43], local triad dynamics and constrained triad dynamics, respectively), and the approach [44]—as [αβα]. In subsequent works, both groups identified both approaches as dealing with the same phenomenon. Other authors added a new cell to the related chain: the presence (β) of thermal noise, as contrasted with the absence of it (α) [45]. With this modification, the approach [44] is encoded as [αβαα], and so on.

The dependence of the diversity, as measures by <*s*>, on the string length *F* shows two opposing effects. With the condition *dc* = *F*, we have to compare the second and sixth line of Table 1. As we see, when *F* increases from 3 to 4, <*s*> is reduced twice (from 0.077 to 0.032). This is the result of the fact that with longer strings, entirely different states are more rare, and the system is more prone to unify. On the other hand, the comparison of the second and fifth lines of Table 1 shows that with the same values of *q* and *dc*, there is just more states of the strings, then both <*s*> and *Smax* increases.

[D] Wave-particle duality is an experimental phenomenon which is visible in different measurements and with varying clarity. A string of cells with wave characteristics (wavelength, frequency) could be supplemented with a string of cells featuring the characteristics of particles (momentum, energy). However, the same states show these or other aspects. Therefore, both descriptions are equivalent, just particular symbols receive a wider interpretation. The entropy is not changed.

[E] In this case, a large set of ideas is unified by means of a sequence of decisions, which themselves were shaped by historical circumstances. Meaningful at the time of their inception, after two millennia some of these ideas seem accidental. For instance, the Orthodox Catholic Church rejects the idea of purgatory accepted by Roman Catholics. The schism in question, irreversible only for political reasons, shows that the actual state in the sequence could have been different. (Despite of this, any religious doctrine claims to be non-modifiable.) Hence, diversity of possible options should be positive. On the other hand, the length *F* of the list of issues which can be meaningful is particularly difficult to be determined.

[F] In the case of quantum mechanics, t* represents the time of publication [27], where the two formulations were shown to be equivalent. We are inclined to treat them as complementary. The question is if there is a place for diversity. According to Steven Hawking, there is no place in physics for ultimate truth [46]. Are there yet more formulations which could serve as alternatives? This option cannot be excluded.

[G] Language is a complex system and entropy is only one of numerous tools to apply there [47]. However, it is straightforward to define entropy for some subset of words and observe how it evolves in time. Apart from the differences between English in USA and UK, splits have been identified between particular regions of USA in frequencies of babies’ names. At the end of XX, these splits gradually disappeared [48].

These arguments indicate that the role of randomness in the dynamics of ideas should not be overlooked. The fact that the works of Todorov [17] and Heider [19] happened to be lying on the bookshelf of Colin Martindale [18] influenced the final outcome of the latter’s research. We could view Johann Eck’s skills as a polemicist as an accidental factor; on the other hand, acceptance of Luther’s theses by the Catholic authorities appears inconceivable. The varied opinions expressed in Catholic synods and the authority they enjoyed in the early Middle Ages, as well as the tangled dynamics of the English language on both sides of the Atlantic are processes too complex to be captured within any deterministic picture. The two other processes can be treated as deterministic if we accept the belief that quantum mechanics is simply the ultimate truth. If not, both examples reflect no more than particular rungs on the ladder of human thought. The garbage can model suggests that other ladders may have been possible.

We do not propose a method that would enable us to evaluate the possible richness of other such ladders—this would be a construction of all alternative histories of human thought. Our way of calculating the diversity of stationary solutions is a metaphor for this task. These results are collected in Table 1. To comment on the role of particular parameters, we argue that the influence exerted by the number of options *q* is understandable if we assume that all states in a string are equally probable. In this case, formula *S* = *Smax* is accurate. Parameters *F* and *dc* should be discussed simultaneously; actually, we should compare the pairs {*F*, *dc* = *F*} and {F, *dc* = *F* − 1} separately. For *dc* = *F*, all distances less than *F* are reduced by the rules of the algorithm. For *dc* = *F* − 1, more different stationary states are possible, and entropy <*s*> is greater.

There is some correspondence between the dynamics of ideas, as discussed here, and the concept of semantic networks [49,50]. On the one hand, semantic networks are useful to represent a spectrum of complex cognitive structures, from words to web pages. To investigate them with tools of statistical mechanics, as entropy, although seemingly straightforward, is rare, with [51] as an exception. Our task here is more limited, as we deal with one particular process—a fusion or a lasting split. On the other hand, an idea in our approach is an object more general than a network node; it has its own internal structure and history, and a network represents a set of its possible states.

## 5. Conclusions

The examples, provided in Section 2 and repeated in Section 4, are discussed here in terms of mathematical models. This is by no means a statement that complex historical phenomena as the evolution of a religious doctrine or a scientific theory can be reduced to a chain of symbols. It is only a certain aspect of time evolution of the related ideas which is in the scope of our considerations. Paying the price of this limitation, still we are left with a quite general perspective.

To conclude, an attempt is made to assign one of two models—the Krause–Hegselmann model and the Axelrod model—to the time evolution of some exemplary ideas. In the latter, randomness is an essential feature; in this way we highlight that the evolution of human thought is not deterministic. Karl Popper [52] expressed this truth tens of years ago in more general terms, in the form of an elaborate essay. Our goal, which according to our knowledge is new, is the construction of a computational illustration of this statement.

## Figures and Tables

**Table 1:** The average value of normalized entropy <*s*>, calculated over single trajectories, taking into account parameters *F*, *q* and *d*_c_. The states are stored for time t* = 10^6^ time steps. For completeness, the last column is added to show normalization constant *Smax*.

*F*	*q*	*dc*	<*s*>	*F* ln*q*
3	4	2	0.460	4.159
3	4	3	0.077	4.159
3	5	2	0.474	4.828
3	5	3	0.099	4.828
4	4	3	0.174	5.545
4	4	4	0.032	5.545
4	5	3	0.208	6.438
4	5	4	0.035	6.438
